# Transactions of the Pathological Society of Philadelphia

**Published:** 1839-10-26

**Authors:** 


### TRANSACTIONS OF THE PATHOLOGICAL SOCIETY OF PHILADELPHIA.

*October 28th,* 1839.

The President, Dr. Gerhard, in the chair.

*Case of Tubercular Meningitis, with softening around the ventricles of the brain—Large cavity in the summit of the right lung, and deposit of gray granulations in the left lung.* By George J. Halson, M. D.

A coloured man, æt. twenty-five, admitted into the Philadelphia Hospital, October 14th, 1839. The anterior history of the patient was not obtained, except that he had had a cough for some months. Upon his entrance there was no symptom of an acute disease; a large cavity was discovered beneath the right clavicle.

On the night of the 16th, two days after entrance, he was attacked with a vomiting of blood, probably flowing into the stomach from the cavity; he vomited nearly a quart of blood, which was arrested by the acetate of lead; immediately after the cessation of the hæmorrhage, he complained of great pain in his chest and abdomen, extending down the thighs, which was relieved by cataplasms of mustard, and he shortly afterwards slept. Next morning he was found in a state of insensibility, with complete paralysis of the right side; mouth drawn to the left; pupils dilated; stertorous breathing. He continued in this state till the morning of the 19th, when, after the effect of a blister, purge, and stimulating enemata, he was enabled to answer questions slowly and indistinctly.

20*th.* Considerable increase of cerebral symptoms; great stupor—cannot be roused; stertorous breathing; puffing of lips; rigidity of both arms and legs, particularly the left; mouth drawn to left side; pupils dilated, insensible to light; subsultus extreme; inability to swallow; *pulse* slow and feeble; respiration high and laboured.

21*st* and 22*d.* No change of symptoms.

23*d.* Rigidity of left arm much diminished,—augmented in right,—most marked in right leg; right eyelid most open; mouth drawn to right side; strong dilatation of nostrils; pulse small and feeble; pupils still dilated.

Died October 24th.

*Necroscopy thirty hours after death.—Brain.* Strong adhesions between dura mater and membrane beneath; two ounces of limpid fluid issued from cavity of arachnoid; the summit of the brain is brightly injected in the finer vessels on both sides; the membranes are opaque in the anterior three-fourths, milky, much thickened; this appearance extends to portions of the membrane between the hemispheres, on one side of which is a regularly formed tubercle, producing a depression in the brain; the substance of the brain is firm and healthy, except immediately contiguous to the ventricles; the ventricles themselves are distended with fluid, which issued in a stream on puncturing the right; quantity unknown; central parts much softened, pulpy, and the internal membrane brightly injected. Bright red injection of the base of the brain; membrane not thickened at posterior part; but from the anterior extremity of the pons varolii is a deposit of lymph beneath the arachnoid, following up the fissures of Sylvius on either side, but most abundant on the left; appearance of distinct granulations; the cerebellum is firm, as well as the rest of the brain.

*Lungs.* The right lung is strongly adherent throughout; the summit of the upper lobe is occupied by a cavity, including nearly its whole extent,—very anfractuous,—filled with a dark-coloured, sanious liquid, traversed by bands; the rest of the lobe is filled with numerous gray granulations; the lower lobe contains gray granulations similar to the upper,—is congested and friable; the bronchial tubes are intensely red and ulcerated. The left lung is soft, permeable to air, offering but few adhesions; numerous gray granulations throughout; the lower lobe is hepatised at the posterior portion; the tissue is very friable and granulated; in this hepatised portion are numerous very minute purulent deposits, with difficulty distinguished from the gray granulations.

Upon the right side of the trachea, opposite the thyroid cartilage, is a sac containing a plastic, caseous deposit, evidently tuberculous.

[This case of tuberculous meningitis is not one of the most frequent kind. Its attack was so sudden, that it resembled very closely a case of ordinary effusion of blood from apoplectic hæmorrhage, which would have been extremely singular in a patient in the last stage of pulmonary consumption. The paralysis was moderate, and extended in unequal and varying degrees to both sides of the body, so that it became evident that the affection was seated in both hemispheres of the brain. The patient presented, the day after the attack, some signs of inflammation, which rather increased before his death.

The lesions were evidently of two kinds: one more permanent and inflammatory, consisting in the injection of the membranes of the exterior of the brain, and of the ventricles; and the second, more rapid and sudden, being rather the effect of inflammation, than this process itself. The first set of lesions consisted not only in the redness of the membranes, but also in the deposits of lymph and of tuberculous granulations scattered throughout it. These probably dated from an earlier period than the paralysis, but at first were latent; the paralysis occurred simultaneously with the effusion of serum, and may be considered as the immediate consequence of the pressure upon the cerebral mass.

The connection between the disease of the brain and the tuberculous diathesis is in this case quite evident, for the patient had long laboured under an aggravated form of phthisis; but in other cases the link is lost, because the meningitis is either the first tuberculous affection, or occurs very early in the course of phthisis. In children it is sometimes still more difficult to trace this connection, because the lungs are less frequently and less early attacked with tuberculous disease. This form of meningitis is, however, much more frequent in children than in adults; and although its origin is traced with some difficulty, it evidently depends upon the general tuberculous diathesis, and the inflammation of the membranes of the brain coincides with the formation of tubercle.

The same law may be discovered in other serous membranes where tuberculous deposits are extremely frequent, and are almost always attended with an inflammatory action. A double secretion results,—first, of the ordinary lymph, pus, and serum; and, secondly, of tubercles in the serous tissue, or inclosed in the false membranes.

In all such cases the tubercles, if seen at an early stage, are found to be surrounded by a well defined ring of newly formed vessels. EDS.]

*Case of Keloides.* By JAS. W. KERR, M. D.

A specimen of keloides, which was removed by an operation by Dr. E. Peace, was then presented by Dr. Kerr, with the following sketch of the case:

M. A., coloured, æt. about twenty-five, single. Has been in the almshouse for two years, during which time she has had two healthy children. She has always enjoyed good health; has never had syphilis. Twenty-one months ago she observed a small tumour on her right side, about the middle of the sixth rib, without pain, which continued to increase in size until "last summer," when she first felt a slight pain in it, which has continued ever since, having become more severe within the last three months, increasing with the growth of the tumour. During the last summer she has not been able to lie on her right side. The pain has always been confined to the tumour.

The tumour is now one inch in length, half an inch in width, and elevated one-eighth of an inch above the surrounding surface; rounded at its extremities. On the surface of the tumour, which is wrinkled, there are a number of depressions which will contain a pin's head, which are filled with a hard, black substance. The intermediate spaces are smooth, shining, and of a somewhat lighter colour than the healthy tissue around the tumour. The tumour offers the feel and resistance of common India-rubber. It arises directly from the surface of the surrounding tissue.

There is another above the spine of the right scapula, half an inch in length, one-eighth of an inch in breadth, and elevated about one-eighth of an inch, which appeared three months ago; she has never felt any pain in it. This one is also increasing in size, and the patient says resembles the larger one when she first observed it. It is smooth and glossy on the surface; contains but two very small depressions, filled with the same black substance as the first tumour, and does not rise so directly from the surface.

There is another of nearly the same size, about the middle of the deltoid muscle of the right arm. Three on the right mamma, and one on the left. All of these have appeared within the last three months. They vary in size; but all present the same smooth, shining surface, without depression, and arising very gradually to a point in their centres, from the surrounding tissue. They are not painful.

The largest tumour was removed on Friday last, by Dr. Peace. The tissue from which the tumour was taken was perfectly healthy.

A report of a series of experiments upon the action of the heart in animals, was then read by Dr. Pennock. This report comprises much interesting and important matter, and will be given in one of our subsequent numbers.

